# Locomotion changes in methamphetamine and amphetamine withdrawal: a systematic review

**DOI:** 10.3389/fphar.2024.1428492

**Published:** 2024-07-17

**Authors:** Jaya Kumar, Isa Naina Mohamed, Rashidi Mohamed, Azizah Ugusman, Mustapha Muzaimi, Wael Mohamed, Mohamad Fairuz Yahaya, Seong Lin Teoh, Mohammad Rahim Kamaluddin, Hafizah Abdul Hamid, Muhammad Zulfadli Mehat, Prem Kumar Shanmugam

**Affiliations:** ^1^ Department of Physiology, Faculty of Medicine, Universiti Kebangsaan Malaysia, Kuala Lumpur, Malaysia; ^2^ Department of Pharmacology, Faculty of Medicine, Universiti Kebangsaan Malaysia, Kuala Lumpur, Malaysia; ^3^ Department of Family Medicine, Faculty of Medicine, Universiti Kebangsaan Malaysia, Kuala Lumpur, Malaysia; ^4^ Department of Neurosciences, School of Medical Sciences, Universiti Sains Malaysia, Kota Bharu, Malaysia; ^5^ Basic Medical Science Department, Kulliyyah of Medicine, International Islamic University Malaysia, Kuantan, Malaysia; ^6^ Department of Clinical Pharmacology, Faculty of Medicine, Menoufia University, Shebin El Kom, Egypt; ^7^ Department of Anatomy, Faculty of Medicine, Universiti Kebangsaan Malaysia, Cheras, Malaysia; ^8^ The Centre for Research in Psychology and Human Well-Being, Faculty of Social Sciences and Humanities, The National University of Malaysia, Bangi, Malaysia; ^9^ Department of Human Anatomy, Faculty of Medicine and Health Sciences, Universiti Putra Malaysia, Selangor, Malaysia; ^10^ Manchester Metropolitan University, Kota Kinabalu, Malaysia

**Keywords:** methamphetamine, amphetamine, stimulant, locomotion, withdrawal, abstinence, systematic review, addiction

## Abstract

Despite extensive preclinical research over the years, a significant gap remains in our understanding of the specific effects of methamphetamine (METH) and amphetamine (AMPH) withdrawal. Understanding these differences could be pivotal to unveiling the unique pathophysiology underlying each stimulant. This may facilitate the development of targeted and effective treatment strategies tailored to the specific characteristics of each substance. Following PRISMA guidelines, this systematic review was conducted to examine alterations in spontaneous locomotor activity, specifically horizontal activity, in animals experiencing withdrawal from extended and repeated administration of AMPH or METH. Original articles were retrieved from four electronic databases, supplemented by a review of the references cited in the published papers. A total of thirty-one full-length articles (n = 31) were incorporated in the analysis. The results indicated that six studies documented a significant increase in horizontal activity among animals, seven studies reported decreased locomotion, and eighteen studies (8 AMPH; 10 METH) reported no significant alterations in the animals’ locomotor activity. Studies reporting heightened locomotion mainly employed mice undergoing withdrawal from METH, studies reporting diminished locomotion predominantly involved rats undergoing withdrawal from AMPH, and studies reporting no significant changes in horizontal activity employed both rats and mice (12 rats; 6 mice). Drug characteristics, routes of administration, animal models, dosage regimens, duration, and assessment timing seem to influence the observed outcomes. Despite more than 50% of papers enlisted in this review indicate no significant changes in the locomotion during the stimulant withdrawal, the unique reactions of animals to withdrawal from METH and AMPH reported by some underscore the need for a more nuanced understanding of stimulant withdrawal.

## 1 Introduction

The global prevalence of psychostimulant use, specifically methamphetamine (METH) and amphetamine (AMPH), has been on the rise (Bach et al., 2023; Castaldelli-Maia et al., 2023). Despite extensive exploration of different compounds and substances, there remains a lack of approved therapeutic agents for stimulant use disorder (Chan et al., 2019; Hazani et al., 2022). Withdrawal from prolonged stimulant use in humans is characterized by symptoms such as dysphoric mood, fatigue, sleep disturbances, increased appetite, and psychomotor changes (Zhao et al., 2021). In rodent studies, various pre-treatment regimens have been employed to replicate abstinence-related symptoms observed in humans, including anhedonia, anxiety, depression, stereotyped behavior, cognitive deficits, and psychomotor changes (Robinson and Camp, 1987; González et al., 2014; Mouton et al., 2016).

Alterations in movement serve as a sensitive indicator of the neurochemical and behavioral changes associated with drug dependence, revealing the positive and negative reinforcing effects of drugs. Drugs of abuse, through elevation of dopamine levels in the nucleus accumbens, mediate positive reinforcing effects of drugs, which initiates drug-seeking behavior. In contrary, negative reinforcement, creates a strong urge in drug-dependent individuals continue to use drugs to avoid drug withdrawal-associated adverse experience. Several locomotor changes are commonly studied in rodents within the context of drug dependence research, each providing valuable insights into the direct impact of drugs and withdrawal on the brain and behavior such as horizontal activity (distance travelled), vertical activity (rearing), and movement patterns (Iman et al., 2021). The alterations in locomotion observed during withdrawal from repeated pre-treatment with AMPH or METH can provide insights into various aspects of neurobehavior, depending on the changes observed. These changes may indicate behavioral sensitization to repeated dosing of the drug (Nakagawa et al., 2011), the presence of abstinence-related dysphoric symptoms (Kitanaka et al., 2012), disruption of sensorimotor gating (Richetto et al., 2013), heightened levels of stress or anxiety (Bray et al., 2016), and reduction in dopaminergic function (Kitanaka et al., 2008).

The goal of this systematic review was to examine current research on alterations in spontaneous locomotor activity, with a specific emphasis on horizontal activity in animals undergoing withdrawal from extended repeated pre-treatments with AMPH or METH.

## 2 Methods

### 2.1 Search strategy

The systematic review included data from four online databases such as SCOPUS, Web of Science, PubMed, and Ovid MEDLINE spanning from 1946 to December 2023, with the latest search conducted on 1 December 2023. The basic search strategy involved combining keywords as follows: (amphetamine OR methamphetamine) AND (abstinence OR withdrawal) AND (locomotion). Further studies were performed by reviewing the references in the retrieved articles.

### 2.2 Inclusion criteria

All full-length research articles published in English that investigated changes in animals’ spontaneous locomotor activity during withdrawal from repeated administrations of AMPH or METH were included.

### 2.3 Exclusion criteria

Case studies, case series, letters to editors, reviews, books, human studies, cell culture studies, and conference abstracts were excluded. Additionally, animal studies focusing on the acute or chronic effects of METH or AMPH without examining the withdrawal, or the impact of an acute AMPH or METH challenge on locomotor sensitization, were excluded. Studies that explored AMPH or METH withdrawal in animals without comparing locomotor differences between control animals (or baseline values) and withdrawn animals were also excluded. Studies involving animals that underwent brain surgery (e.g., microinfusion of drugs, intracranial stimulation, and brain lesions) were not considered. Furthermore, studies examining METH or AMPH withdrawal in animals that had undergone surgery or behavioral interventions (such as sleep deprivation, fear conditioning, and encounters with intruders) prior to drug pre-treatment were omitted. Studies administering other drugs (such as cocaine, heroin, alcohol, and caffeine) before METH or AMPH intake were also not included.

### 2.4 Study selection and article screening

Four authors (INM, RPMP, AU, and JK) independently reviewed the articles obtained from the databases. Any discrepancies were resolved through discussion to achieve a consensus. The article screening process comprised of three stages. Initially, titles were used as the basis for rejecting articles that did not meet selection criteria. Subsequently, the abstracts were reviewed to eliminate studies unrelated to AMPH or METH withdrawal and locomotion. Finally, a comprehensive examination of the full text was conducted to exclude articles that did not meet the inclusion criteria.

## 3 Results

**FIGURE 1:**
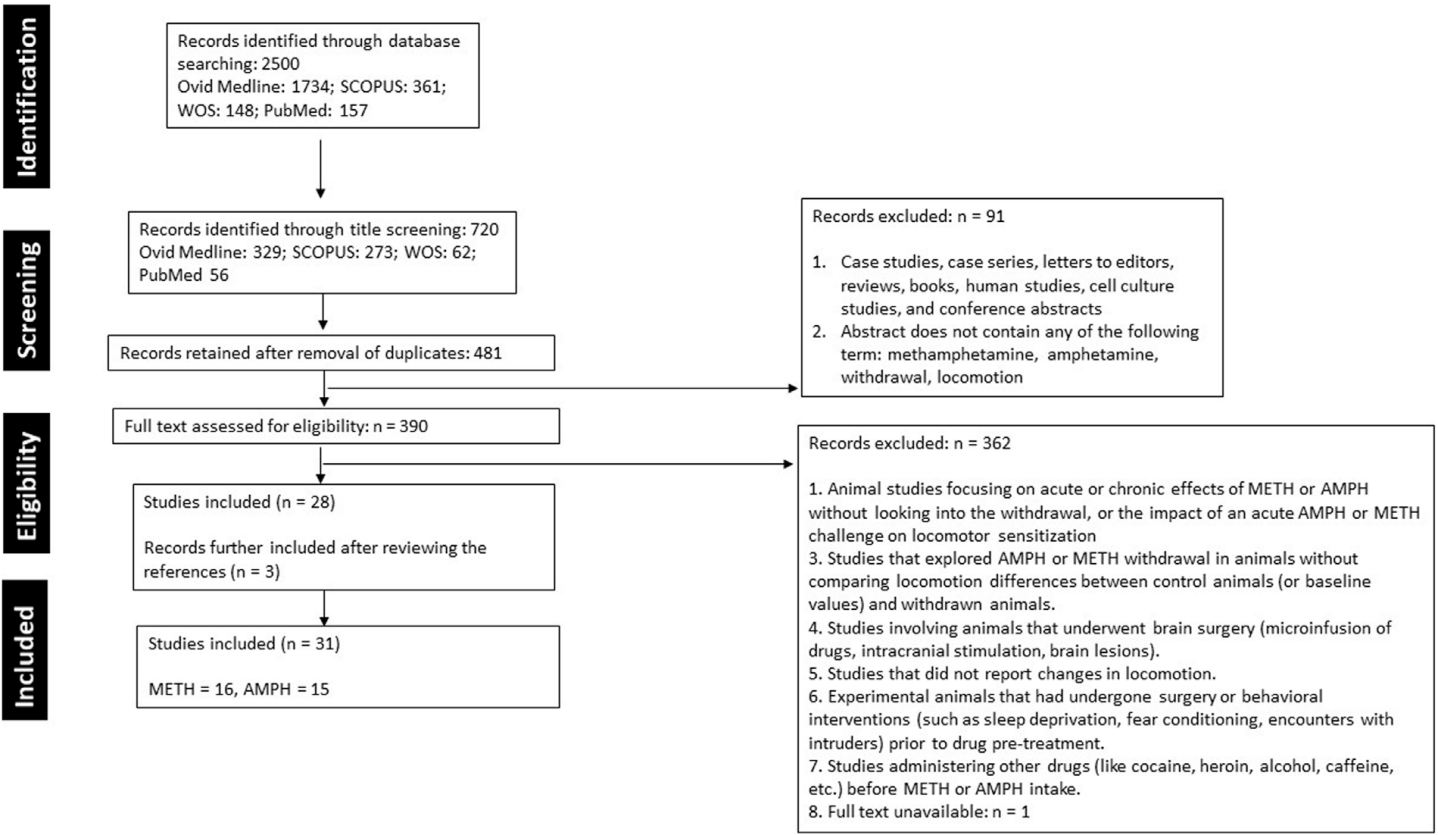
A summary of the literature search, screening, and selection of studies following the Preferred Reporting Items for Systematic Reviews and Meta-Analyses (PRISMA) guidelines.

Initially, 2,500 articles were identified across four online databases: Ovid MEDLINE (1,734), SCOPUS (461), Web of Science (148), and PubMed (157). Through title screening, 720 articles were identified (Ovid MEDLINE: 329, SCOPUS: 273, Web of Science: 62, PubMed: 56). After the removal of duplicates, 481 articles remained. This was followed by a rigorous review of the abstracts, methods, and results based on the inclusion criteria, resulting in the rejection of 452 articles. Ultimately, 28 original, full-length articles were included. Additionally, upon reviewing the references of these articles, an additional 3 articles were added, bringing the final count of selected articles to 31 (Figure 1).

Of the 31 studies included in this systematic review, 16 used METH (methamphetamine hydrochloride, (+)-methamphetamine, and d-methamphetamine), whereas 15 investigated AMPH (d-amphetamine sulfate, d-amphetamine), mostly dissolved in normal saline. The drugs were mainly given via intraperitoneal (24 studies), subcutaneous (4), and intravenous routes (1), mixed with tap water when taken orally (1), or inhaled (1).

Eleven studies employed mice, mostly male (9 studies), female only (1; Haidar et al., 2016), and both male and female (1; Henry et al., 2013). Twenty studies employed rats, mostly male (17 studies), female only (2; Paulson et al., 1991; Robinson and Camp, 1987), and both male and female (1; Mouton et al., 2016). Various strains of mice were used, including C57BL/6J (4 studies), BALB/c (1; Ghavimi et al., 2022), NMRI (2; Haj-Mirzaian et al., 2018; Hosseini et al., 2021), Rln KO, Rfxp3 KO mice (1; Haidar et al., 2016), CD1 (1; Mandillo et al., 2003), gp120tg (1; Henry et al., 2013), and strain type not stated (1; Roohbakhsh et al., 2021). Likewise, different strains of rats were used, such as Wistar (12 studies), Sprague Dawley (5), Holtzman (1; Paulson et al., 1991), FSL and FRL (1; Mouton et al., 2016), and long Evans (1; Sharma et al., 2021).

The number of animals employed per group in the studies were N = 4–6 (1 study), 5–6 (1), 6 (1), 6–7 (2), 7 (1), 7–8 (1), 6–8 (3), 8 (3), 8–9 (1), 7–10 (2), 8–14 (1), 9 (1), 10 (3), 11 (1), 12–13 (1), 11–14 (1), 19–22 (1), and not mentioned (6).

Of the reviewed studies, six studies (total n = 6; n = 5 METH, n = 1 AMPH) reported a significant increase in the animals’ horizontal activity (Piechota et al., 2012; Georgiou et al., 2016; Haidar et al., 2016; Haj-Mirzaian et al., 2018; Rezaeian et al., 2020; Roohbakhsh et al., 2021), seven studies (total n = 7; n = 6 AMPH, n = 1 METH) reported significant decrease in locomotor activity (Robinson and Camp, 1987; Paulson et al., 1991; Pulvirenti and Koob, 1993; Hsieh et al., 2002; Russig et al., 2005; Che et al., 2013; Mouton et al., 2016) and 18 studies (total n = 18; n = 8 AMPH, n = 11 METH) reported no significant changes in the animals’ locomotion (Persico et al., 1995; Cancela et al., 2001; Mandillo et al., 2003; Russig et al., 2005; Peleg-Raibstein et al., 2006; Barr et al., 2010; Henry et al., 2013; Koltunowska et al., 2013; González et al., 2014; Damghani et al., 2016; Marszalek-Grabska et al., 2016; Beirami et al., 2017; Saeed et al., 2018; Alavijeh et al., 2019; Yan et al., 2019; Hosseini et al., 2021; Sharma et al., 2021; Ghavimi et al., 2022) (Table 1).

**TABLE 1:** Summary of included studies.

No	References	Druxg	Findings
Hyperlocomotion during withdrawal			
1	Rezaeian et al. (2020)	METH	Increased total distance travelled, increased distance travelled in the peripheral zone, decreased distance travelled in the central zone
2	Georgiou et al. (2016)	METH	Increased total distance travelled, and rearing
3	Haidar et al. (2016)	METH	Increased total distance travelled by Rln3 WT on D9 of withdrawal
4	Roohbakhsh et al. (2021)	METH	Increased total square crossing and square crossing in centre of open field
5	Piechota et al. (2012)	METH	Increased beam crossing
6	Haj-Mirzaian et al. (2018)	AMPH	Increased total distance travelled, and number of rearings
Hypolocomotion during withdrawal			
7	Mouton et al. (2016)	METH	Reduced total distance travelled on PostND35, but not on PostND60
8	Che et al. (2013)	AMPH	Withdrawal from 4 mg/kg AMPH: Decreased distance travelled, time in the center, number of rearing, and frequency of center entries Withdrawal from 2 mg/kg AMPH: Decreased frequency of center entries only
9	Pulvirenti and Koob (1993)	AMPH	Reduced spontaneous locomotor activity
10	Russig et al. (2005)	AMPH	Group ESC-10: Reduced locomotor activity
11	Hsieh et al. (2002)	AMPH	Withdrawal D3: Reduced locomotor activity count
12	Paulson et al. (1991)	AMPH	Withdrawal day 2–3: Reduced locomotor activity (crossovers) on both day and night Withdrawal day 4–7: Reduced locomotor activity at night
13	Robinson and Camp (1987)	AMPH	Reduced activity counts during night-time
No significant changes in locomotion			
14	Henry et al. (2013)	METH	No changes in total distance travelled
15	Alavijeh et al. (2019)	METH	No changes in total distance travelled Decreased time spent, crossings produced in the central area of the open field
16	Beirami et al. (2017)	METH	No difference in total number of beam breaks
17	Yan et al. (2019)	METH	No difference in total distance travelled
18	Gonzalez et al. (2014)	METH	No difference in total distance travelled
19	Sharma et al. (2021)	METH	No difference in total distance travelled
20	Ghavimi et al. (2022)	METH	No difference in total distance travelled
21	Hosseini et al. (2021)	METH	No difference in total distance travelled
22	Damghani et al. (2016)	METH	No difference in swimming velocity
23	Saeed et al. (2018)	METH	No difference in swimming speed
24	Russig et al. (2003)	AMPH	No difference in the baseline activity levels (breakpoints)
25	Persico et al. (1995)	AMPH	No difference in total distance travelled
26	Marszalek-Grabska et al. (2016)	AMPH	No difference in total distance travelled
27	Peleg-Raibstein et al. (2006)	AMPH	No difference in baseline locomotor activity (activity score)
28	Mandillo et al. (2003)	AMPH	No difference in locomotor activity, grooming, and leaning behaviors Reduced rearing in 2.5 mg/kg AMPH withdrawal
29	Koltunowska et al. (2013)	AMPH	No difference in distance travelled
30	Cancela et al. (2001)	AMPH	No difference in total arm entries
31	Barr et al. (2010)	AMPH	No difference in total distance travelled

### 3.1 Hyperlocomotion during the abstinence

Studies reporting a significant increase in the animals’ locomotion during the withdrawal period mainly came from mice as experimental subjects (5 studies) and to a lesser extent, rats (1 study). In five out of six of these studies, METH was administered, and most of these studies (5 studies) used the intraperitoneal route as mode of drug administration, except for one study in which animals inhaled the drug (Rezaeian et al., 2020).

The drug administration period was ranging from 1 day (10 mg/kg METH i. p, 4 times per day; Roohbakhsh et al., 2021), 5 days (5 mg/kg AMPH i. p; Haj-Mirzaian et al., 2018), 10 days (2 mg/kg METH i. p; Georgiou et al., 2016; escalating dose 2–6 mg/kg METH i. p; Haidar et al., 2016), 12 days (escalating dose 2–8 mg/kg METH i. p, twice daily; Piechota et al., 2012) to 14 days (inhaled: first week 5 mg/kg, second week 10 mg/kg; Rezaeian et al., 2020) (Table 2).

**TABLE 2:** Increased locomotion during the withdrawal period: Drug administration regimen.

References	Strain/Species	Gender	Age	Drug	Duration of drug administration	Frequency of administration	Dosage	Mode of administration	Animal/group
Rezaeian et al. (2020)	Rat Wistar	Male	-	METH	14 days	1 time	W1–5 mg/kg W2- 10 mg/kg	inhaled	9
Georgiou et al. (2016)	Mice C57BL/6J	Male	8 weeks	METH	10 days	1 time	2 mg/kg	i.p	-
Haidar et al. (2016)	Mice Rln3KO	Female	8–10 weeks	METH	10 days	1 time	ESC 2–6 mg/kg	i.p	8–14
Roohbakhsh et al. (2021)	Mice	Male	-	METH	1 day	4 times 2 h interval	10 mg/kg	i.p	6
Piechota et al. (2012)	Mice C57BL/6J	Male	8–10 weeks	METH	12 days	2 times	ESC 2–8 mg/kg	i.p	4–6
Haj-Mirzaian et al. (2018)	Mice NMRI	Male	-	AMPH	5 days	1 time	5 mg/kg	i.p	6–8

Significant changes in the locomotion of drug withdrawn animals were reported on withdrawal day 1 (Haj-Mirzaian et al., 2018; Roohbakhsh et al., 2021), day 7 (Georgiou et al., 2016), day 9 (Haidar et al., 2016), day 12 (Piechota et al., 2012) and day 22 (Rezaeian et al., 2020). Three studies conducted the behavioral assessment during the light phase of the day: Rezaeian et al. (2020) used 0800–15.00, Georgiou et al. (2016) used 0800, Haidar et al. (2016) used 0900–17.00, and 3 studies did not specify the time. The duration of the behavioral assessment varied from 5 (Haj-Mirzaian et al., 2018; Rezaeian et al., 2020), 10 (Roohbakhsh et al., 2021), 60 (Piechota et al., 2012; Haidar et al., 2016), to 90 min (Georgiou et al., 2016).

Four of these studies assessed the distance travelled to measure the animals’ horizontal activity (Georgiou et al., 2016; Haidar et al., 2016; Haj-Mirzaian et al., 2018; Rezaeian et al., 2020) and the remaining two assessed total square crossing (Roohbakhsh et al., 2021) and beam crossing (Piechota et al., 2012) using behavioral apparatus such as plexiglass box, open field box, locomotion chamber, locomotor cell, wooden cage, and test cage with photocells (Table 3).

**TABLE 3:** Increased locomotion during the withdrawal period: Behavioral assessment.

References	Light/dark phase	Duration of assessment (min)	Withdrawal day	Behavioral apparatus	Parameters used
Rezaeian et al. (2020)	Light	5	D22	Open field	Distance travelled
Georgiou et al. (2016)	Light	90	D7	Locomotion chamber	Distance travelled
Haidar et al. (2016)	Light	60	D9	Locomotor cell	Distance travelled
Roohbakhsh et al. (2021)	-	10	D1	Wooden cage	Square crossing
Piechota et al. (2012)	-	60	D12	Test cage with photocells	Beam crossing
Haj-Mirzaian et al. (2018)	-	5	D1	Open field	Distance travelled

The allocation of animals per group was ranging from to 4–6 (Piechota et al., 2012), 6 (Roohbakhsh et al., 2021), 6–8 (Haj-Mirzaian et al., 2018), 8–14 (Haidar et al., 2016), 9 (Rezaeian et al., 2020), and one study did not state the number (Georgiou et al., 2016).

### 3.2 Hypolocomotion during the abstinence

In contrast to hyperlocomotion results, all studies reporting hypolocomotion during the abstinence period employed rats as their experimental subjects, males (4 studies) and females (3 studies). All these studies administered AMPH, mostly via the intraperitoneal route (5 studies), except for two studies that employed intravenous (Pulvirenti and Koob, 1993) and subcutaneous modes (Mouton et al., 2016).

Drug administration period ranged from 4 days (escalating dose 1–10 mg/kg i. p, thrice daily, except for day 4 where single high dose was given; Russig et al., 2005), 10 days (intravenous self-administration of 0.12 mg/kg per injection; Pulvirenti and Koob, 1993), 14 days (4 mg/kg i. p; Che et al., 2013, 5 mg/kg i. p; Hsieh et al., 2002), 16 days (escalating dose 0.2–6 mg/kg s. c, twice daily; Mouton et al., 2016) and 42 days (escalating dose 1–10 mg/kg i. p, twice daily excluding the weekends; Paulson et al., 1991; Robinson and Camp, 1987) (Table 4).

**TABLE 4:** Reduced locomotion during the withdrawal period: Drug administration regimen.

References	Strain/Species	Gender	Age	Drug	Duration of drug administration	Frequency of administration	Dosage	Mode of administration	Animal/group
Mouton et al. (2016)	Rats FSL and FRL	Female	-	METH	16 days	2 times	ESC 0.2–6 mg/kg	s.c	-
Che et al. (2013)	Rat Sprague Dawley	Male	-	AMPH	14 days	1 time	4 mg/kg	i.p	8
Pulvirenti and Koob (1993)	Rat Wistar	Male	-	AMPH	10 days	-	0.12 mg/kg per infusion Intake: 5.9–9.6 mg/kg	i.v (self-administration)	11–14
Russig et al. (2005)	Rat Wistar	Male	-	AMPH	4 days	3 times (D1-3)	ESC 1–9 mg/kg (D1-3); D4: 10 mg/kg (1X)	i.p	-
Hsieh et al. (2002)	Rat Sprague Dawley	Male	-	AMPH	14 days	1 time	5 mg/kg	i.p	6–8
Paulson et al. (1991)	Rat Holtzmann	Female	-	AMPH	42 days Intermittent (no weekend)	2 times	ESC 1–10 mg/kg	i.p	6–7
Robinson and Camp (1987)	Rat Sprague Dawley	Female	-	AMPH	42 days Intermittent (no weekend)	2 times	ESC 1–10 mg/kg	i.p	7

Significant changes in the horizontal activity of the animals were reported on withdrawal days 1 (Russig et al., 2005), 2 and 4 (Pulvirenti and Koob, 1993) 3 (Hsieh et al., 2002), 6 (Mouton et al., 2016), 8–12 (Robinson and Camp, 1987), 10–14 (Che et al., 2013), and 23–28 (Paulson et al., 1991). Two studies conducted the behavioral assessment during the light phase (Che et al., 2013; Hsieh et al., 2002, 10.00–10.30) and three studies conducted during the dark phase (5p.m.–8 a.m., Mouton et al., 2016; Pulvirenti and Koob, 1993; Russig et al., 2005), and another two during both light and dark phase (21.5 h, Paulson et al., 1991; 20 h; Robinson and Camp, 1987). The last two studies (Robinson and Camp, 1987; Paulson et al., 1991) reported a significant decrease in the horizontal activity of AMPH withdrawn rats during the dark phase of the day. The animals’ behavior was assessed for 5 min (Che et al., 2013; Mouton et al., 2016), 30 min (Hsieh et al., 2002), 60 min (Russig et al., 2005), 180 min (Pulvirenti and Koob, 1993), 20 h (Robinson and Camp, 1987), and 21.5 h (Paulson et al., 1991).

The parameters assessed were distance travelled (Che et al., 2013; Mouton et al., 2016), photobeam interruption (Pulvirenti and Koob, 1993), locomotor activity counts (Hsieh et al., 2002), locomotor activity (Russig et al., 2005), crossovers (Paulson et al., 1991), and activity counts (Robinson and Camp, 1987), using behavioral apparatus such as the open field, wire cages, wooden cabin stations, activity chamber, automated activity monitor, wire hanging cages, and animal activity monitor (Table 5).

**TABLE 5:** Reduced locomotion during the withdrawal period: Behavioral assessment.

References	Light/dark phase	Duration of assessment	Withdrawal day	Behavioral apparatus	Parameters used
Mouton et al. (2016)	Dark	5 min	D6	Animal activity monitor	Distance travelled
Che et al. (2013)	Light	5 min	D10-14	Open field	Distance travelled
Pulvirenti and Koob (1993)	Dark	180 min	D2, D4	Wire cages	Photobeam interruption
Russig et al. (2005)	Dark	60 min	D1	Wooden cabins	Locomotor activity
Hsieh et al. (2002)	Light	30 min	D3	Activity chamber	Locomotor activity counts
Paulson et al. (1991)	Light & Dark	21.5 h	D23-28	Automated activity monitor	Crossovers
Robinson and Camp (1987)	Light & Dark	20 h	D8-12	Wire hanging cages	Activity counts

The number of animals employed per group was 6–7 (1 study), 6–8 (1), 7 (1), 8 (1), 11–14 (1), except for two studies that did not specify the number of animals.

### 3.3 No significant changes in locomotion during the abstinence

Studies that found no significant changes in the locomotion of animals withdrawn from the drug mostly employed rats (12 studies) and to a lesser extent, mice (6 studies). Eight studies administered AMPH via the intraperitoneal route, ten studies administered METH via subcutaneous (3 studies), intraperitoneal (6 studies) and water (Alavijeh et al., 2019).

The drug administration period ranged from 4 days (escalating dose 1–10 mg/kg AMPH i. p thrice daily; Peleg-Raibstein et al., 2006), 5 days (2.5 or 5 mg/kg AMPH i. p; Mandillo et al., 2003), 6 days (escalating dose AMPH 1–5 mg/kg i. p, thrice daily and fixed dosing 1.5 mg/kg intermittent, i. p; Russig et al., 2005), 7 days (1 mg/kg METH i. p, Yan et al., 2019; 1 mg/kg METH s. c; González et al., 2014; 10 mg/kg METH i. p; Saeed et al., 2018), 9 days (2 mg/kg AMPH i. p, Cancela et al., 2001), 10 days (escalating dose 1–10 mg/kg METH i. p twice daily, Beirami et al., 2017; 10 mg/kg METH i. p; Sharma et al., 2021, 14 days (8 studies: escalating dose 20 mg/L to 12 mg/kg METH mixed with water; Alavijeh et al., 2019; 7.5 mg/kg AMPH twice daily; Persico et al., 1995; 2 mg/kg AMPH i. p; Marszalek-Grabska et al., 2016; 2 mg/kg METH i. p, twice daily; Ghavimi et al., 2022; 2 mg/kg METH i. p, twice daily; Hosseini et al., 2021; 2 mg/kg METH s. c, twice daily; Damghani et al., 2016; 2.5 mg/kg AMPH i. p; Koltunowska et al., 2013; 2.5 mg/kg AMPH i. p; Barr et al., 2010, and 25 days (escalating dose 14 days, 0.1–4 mg/kg s. c, thrice daily METH +11 days of 6 mg/kg METH, 4 times per day; Henry et al., 2013) (Table 6).

**TABLE 6:** No significant changes in locomotion during the withdrawal period: Drug administration regimen.

References	Strain/Species	Gender	Age	Drug	Duration of drug administration	Frequency of administration	Dosage	Mode of administration	Animal/group
Henry et al. (2013)	Mice Gp120tg	Male and female	8–9 months 4 months	METH	25 days	3 times	0.1–4 mg/kg (11 days) + 6 mg/kg (4x/day)	s.c	12–13
Alavijeh et al. (2019)	Rat Wistar	Male	-	METH	14 days	1 time	ESC 20 mh/L–12 mg/kg	Drinking water	10
Beirami et al. (2017)	Rat Wistar	Male	Adult	METH	10 days	2 times	ESC 1–10 mg/kg METH	i.p	10
Yan et al. (2019)	Mice C57BL/6J	Male	Adult	METH	7 days	1 time	1 mg/kg	i.p	10
Gonzalez et al. (2014)	Mice C57BL/6J	Male	2–3 months	METH	7 days	1 time	1 mg/kg	s.c	8–9
Sharma et al. (2021)	Rats Long Evan	Male	2 months	METH	10 days	1 time	10 mg/kg	i.p	8
Ghavimi et al. (2022)	Mice BACB/c	Male	-	METH	14 days	2 times	2 mg/kg	i.p	6–7
Hosseini et al. (2021)	Mice NMRI	Male	-	METH	14 days	2 times	2 mg/kg	i.p	6–8
Damghani et al. (2016)	Rat Wistar	Male	Adult	METH	14 days	2 times	2 mg/kg	s.c	7–8
Saeed et al. (2018)	Rat Wistar	Male	-	METH	7 days	1 time	10 mg/kg	i.p	-
Russig et al. (2003)	Rat Wistar	Male	-	AMPH	6 days	3 times 1 time	ESC 1–5 mg/kg 1.5 mg/kg Intermittent	i.p	8
Persico et al. (1995)	Rat Sprague Dawley	Male	-	AMPH	14 days	1 time	7.5 mg/kg	i.p	5–6
Marszalek-Grabska et al. (2016)	Rat Wistar	Male	-	AMPH	14 days	1 time	2 mg/kg	i.p	-
Peleg-Raibstein et al. (2006)	Rat Wistar	Male	-	AMPH	4 days	3 times	ESC 1–10 mg/kg	i.p	-
Mandillo et al. (2003)	Mice CD1	Male	7–9 weeks	AMPH	5 days	1 time	2.5 mg/kg	i.p	7–10
Koltunowska et al. (2013)	Rat Wistar	Male	Adult	AMPH	14 days	1 time	2.5 mg/kg	i.p	7–10
Cancela et al. (2001)	Rat Wistar	Male	-	AMPH	9 days	1 time	2 mg/kg	i.p	11
Barr et al. (2010)	Rat Sprague Dawley	Male	Adult	AMPH	14 days	1 time	2.5 mg/kg	i.p	19–22

Locomotion was assessed at 1 h (Marszalek-Grabska et al., 2016), 1, 6, 12, 24, 36, 54, and 168 h (Persico et al., 1995), day 1 (Koltunowska et al., 2013), day 1–3 (González et al., 2014), day 4 (Cancela et al., 2001; Sharma et al., 2021), day 7 (Henry et al., 2013), day 10 (Ghavimi et al., 2022), day 10–14 (Saeed et al., 2018), day 11 and day 17 (Beirami et al., 2017), day 12 (Hosseini et al., 2021), day 14 (Damghani et al., 2016), day 21 (Alavijeh et al., 2019), day 28 (Barr et al., 2010), day 30 (Russig et al., 2005), day 46, 53, and 60 (Yan et al., 2019), and day 75 (Peleg-Raibstein et al., 2006). Three studies assessed behavior during the dark phase (Russig et al., 2005; Peleg-Raibstein et al., 2006; Barr et al., 2010), seven assessed behavior during the light phase (Mandillo et al., 2003; Henry et al., 2013; González et al., 2014; Marszalek-Grabska et al., 2016; Yan et al., 2019; Hosseini et al., 2021; Ghavimi et al., 2022), and eight studies did not state the time of the behavioral assessment (Schindler et al., 1994; Cancela et al., 2001; Koltunowska et al., 2013; Damghani et al., 2016; Beirami et al., 2017; Saeed et al., 2018; Alavijeh et al., 2019; Sharma et al., 2021). Assessment of animal behavior was conducted for 5 min (7 studies: Alavijeh et al., 2019; Barr et al., 2010; Beirami et al., 2017; Cancela et al., 2001; Ghavimi et al., 2022; González et al., 2014; Hosseini et al., 2021), 1 min (Damghani et al., 2016; Saeed et al., 2018), 6 min (Mandillo et al., 2003), 10 min (Sharma et al., 2021), 15 min (Koltunowska et al., 2013; Marszalek-Grabska et al., 2016), 30 min (Schindler et al., 1994; Henry et al., 2013), 60 min (Russig et al., 2005; Yan et al., 2019), and 18 h (Peleg-Raibstein et al., 2006).

Behavioral parameters such as the distance travelled (11 studies: Alavijeh et al., 2019; Barr et al., 2010; Ghavimi et al., 2022; González et al., 2014; Henry et al., 2013; Hosseini et al., 2021; Koltunowska et al., 2013; Marszalek-Grabska et al., 2016; Schindler et al., 1994; Sharma et al., 2021; Yan et al., 2019), breakpoints (Russig et al., 2005), total number of beam breaks (Beirami et al., 2017), activity scores (Peleg-Raibstein et al., 2006), locomotor activity (Mandillo et al., 2003), swimming velocity (Damghani et al., 2016; Saeed et al., 2018), and total arm entries (Cancela et al., 2001) were measured using behavioral apparatus such as compartment within wooden cabinet, activity monitor, behavioral pattern monitor, open field chamber, Porfex photocell apparatus, circular pool, and elevated plus maze (Table 7).

**TABLE 7:** No significant changes in locomotion during the withdrawal period: Behavioral assessment.

References	Light/dark phase	Duration of assessment	Withdrawal day	Behavioral apparatus	Parameters used
Henry et al. (2013)	Light	30 min	D7	Behavioral pattern monitor	Distance travelled
Alavijeh et al. (2019)	-	5 min	D21	Open field	Distance travelled
Beirami et al. (2017)	-	5 min	D11-17	Open field	Total number of beam breaks
Yan et al. (2019)	Light	60 min	D46, 53, 60	Open field	Distance travelled
Gonzalez et al. (2014)	Light	5 min	D1-3	Open field	Distance travelled
Sharma et al. (2021)	-	10 min	D4	Open field	Distance travelled
Ghavimi et al. (2022)	Light	5 min	D10	Open field	Distance travelled
Hosseini et al. (2021)	Light	5 min	D12	Open field	Distance travelled
Damghani et al. (2016)	-	1 min	D14	Circular pool	Swim velocity
Saeed et al. (2018)	-	1 min	D10-14	Circular pool	Swim speed
Russig et al. (2003)	Dark	60 min	D30	Wooden cabinet	Breakpoint
Persico et al. (1995)	-	30 min	1, 6, 12, 24, 36, 54, 168 h	Activity monitor	Distance travelled
Marszalek-Grabska et al. (2016)	Light	15 min	1 h	Porfex photocell apparatus	Distance travelled
Peleg-Raibstein et al. (2006)	Dark	18 h	D75	Automated behaviour apparatus	Activity scores
Mandillo et al. (2003)	Light	6 min	D1	Open field	Locomotor activity
Koltunowska et al. (2013)	-	15 min	D1	Photocell apparatus	Distance travelled
Cancela et al. (2001)	-	5 min	D4	Elevated plus maze	Total arm entries
Barr et al. (2010)	Dark	5 min	D28	Elevated plus maze	Distance travelled

The number of animals employed per group was 5–6 (1 study), 6–7 (1), 6–8 (1), 7–8 (1), 8 (2), 8–9 (1), 7–10 (2), 10 (3), 11 (1), 12–13 (1), 19–22 (1), and three studies did not mention the number of animals allocated per group.

### 3.4 Hypolocomotion *versus* no changes in locomotion during the abstinence: rats

Studies reporting reduced locomotion employed a longer dosing period ranging from 4 to 42 days (Robinson and Camp, 1987; Paulson et al., 1991; Pulvirenti and Koob, 1993; Russig et al., 2005; Hsieh et al., 2002; Che et al., 2013; Mouton et al., 2016), while those reporting no significant changes had shorter durations, typically between 4 and 14 days (Persico et al., 1995; Cancela et al., 2001; Russig et al., 2003; Peleg-Raibstein et al., 2006; Barr et al., 2010; Koltunowska et al., 2013; Damghani et al., 2016; Marszalek-Grabska et al., 2016; Beirami et al., 2017; Saeed et al., 2018; Alavijeh et al., 2019; Sharma et al., 2021).

The predominant route of administration for both groups was intraperitoneal. Regarding dosages, the studies reporting reduced locomotion mostly utilized escalating dosages (ranging from 1 to 10 mg/kg in 4 studies, 4 days–42 days, 2–3-time injection per day, Mouton et al., 2016; Russig et al., 2005; Paulson et al., 1991; Robinson and Camp, 1987), while fixed dosage regimens typically fell within the range of 4–5 mg/kg (once daily for 14 days, Che et al., 2013; Hsieh et al., 2002).

In contrast, among studies reporting no significant changes, four studies employed escalating dosing regimen (ranging from 1 to 12 mg/kg, Alavijeh et al., 2019; Beirami et al., 2017; Russig et al., 2003; Peleg-Raibstein et al., 2006), and fixed dosages were mostly within the range of 2–2.5 mg/kg administered once daily (Cancela et al., 2001; Barr et al., 2010; Koltunowska et al., 2013; Damghani et al., 2016; Marszalek-Grabska et al., 2016).

The cumulative doses of studies reporting AMPH withdrawal induced hypolocomotion was within the range of 55–356 mg/kg (Robinson and Camp, 1987; Paulson et al., 1991; Pulvirenti and Koob, 1993; Russig et al., 2005; Hsieh et al., 2002; Che et al., 2013), whereas for those reporting no profound changes in the locomotion was 18–210 mg/kg (Persico et al., 1995; Cancela et al., 2001; Russig et al., 2003; Peleg-Raibstein et al., 2006; Barr et al., 2010; Koltunowska et al., 2013; Marszalek-Grabska et al., 2016).

When comparing studies employing escalating doses, those indicating AMPH-induced hypolocomotion assessed behavior during withdrawal days 1–28 (Robinson and Camp, 1987; Paulson et al., 1991; Pulvirenti and Koob, 1993; Hsieh et al., 2002; Che et al., 2013 Che et al., 2013). Conversely, studies revealing no significant changes in locomotion examined behavior on withdrawal 1 h to D75 (Persico et al., 1995; Cancela et al., 2001; Russig et al., 2003; Peleg-Raibstein et al., 2006; Barr et al., 2010; Koltunowska et al., 2013; Marszalek-Grabska et al., 2016) (Table 8).

**TABLE 8:** Hypolocomotion *versus* no changes in movement: rats.

References	Strain/Species	Gender	Drug	Duration of drug administration	Frequency of administration	Dosage	Light/dark phase	Withdrawal day
Hypolocomotion during withdrawal								
Mouton et al. (2016)	Rats FSL and FRL	Female	METH	16 days	2 times	ESC 0.2–6 mg/kg	Dark	D6
Che et al. (2013)	Rat Sprague Dawley	Male	AMPH	14 days	1 time	4 mg/kg	Light	D10-14
Pulvirenti and Koob (1993)	Rat Wistar	Male	AMPH	10 days	-	0.12 mg/kg per infusion Intake: 5.9–9.6 mg/kg	Dark	D2, D4
Russig et al. (2005)	Rat Wistar	Male	AMPH	4 days	3 times (D1-3)	ESC 1–9 mg/kg (D1-3); D4: 10 mg/kg (1X)	Dark	D1
Hsieh et al. (2002)	Rat Sprague Dawley	Male	AMPH	14 days	1 time	5 mg/kg	Light	D3
Paulson et al. (1991)	Rat Holtzmann	Female	AMPH	42 days Intermittent (no weekend)	2 times	ESC 1–10 mg/kg	Light & Dark	D23-28
Robinson and Camp (1987)	Rat Sprague Dawley	Female	AMPH	42 days Intermittent (no weekend)	2 times	ESC 1–10 mg/kg	Light & Dark	D8-12
No significant changes in locomotion during withdrawal								
Alavijeh et al. (2019)	Rat Wistar	Male	METH	14 days	1 time	ESC 20 mh/L–12 mg/kg	-	D21
Beirami et al. (2017)	Rat Wistar	Male	METH	10 days	2 times	ESC 1–10 mg/kg METH	-	D11-17
Sharma et al. (2021)	Rats Long Evan	Male	METH	10 days	1 time	10 mg/kg	-	D4
Damghani et al. (2016)	Rat Wistar	Male	METH	14 days	2 times	2 mg/kg	-	D14
Saeed et al. (2018)	Rat Wistar	Male	METH	7 days	1 time	10 mg/kg	-	D10-14
Russig et al. (2003)	Rat Wistar	Male	AMPH	6 days	3 times 1 time	ESC 1–5 mg/kg 1.5 mg/kg Intermittent	Dark	D30
Persico et al. (1995)	Rat Sprague Dawley	Male	AMPH	14 days	1 time	7.5 mg/kg	-	1, 6, 12, 24, 36, 54, 168 h
Marszalek-Grabska et al. (2016)	Rat Wistar	Male	AMPH	14 days	1 time	2 mg/kg	Light	1 h
Peleg-Raibstein et al. (2006)	Rat Wistar	Male	AMPH	4 days	3 times	ESC 1–10 mg/kg	Dark	D75
Koltunowska et al. (2013)	Rat Wistar	Male	AMPH	14 days	1 time	2.5 mg/kg	-	D1
Cancela et al. (2001)	Rat Wistar	Male	AMPH	9 days	1 time	2 mg/kg	-	D4
Barr et al. (2010)	Rat Sprague Dawley	Male	AMPH	14 days	1 time	2.5 mg/kg	Dark	D28

### 3.5 Hyperlocomotion *versus* no changes in locomotion during the abstinence: mice

Both sets of studies encompassed various strains or genotypes of mice, including gp120tg, C57BL/6J, BALB/c, NMRI, CD1, and BALB/c, C57BL/6J, and Rln3KO (Georgiou et al., 2016; Haidar et al., 2016; Roohbakhsh et al., 2021; Piechota et al., 2012; A Haj-Mirzaian et al., 2018; Henry et al., 2013; Yan et al., 2019; Gonzalez et al., 2014; Ghavimi et al., 2022; Hosseini et al., 2021; Mandillo et al., 2003).

Studies reporting hyperlocomotion following METH withdrawal had dosing regimens lasting from 1 to 12 days (Piechota et al., 2012; Georgiou et al., 2016; Haidar et al., 2016; Roohbakhsh et al., 2021). Among these, two studies utilized escalating dosages (2–8 mg/kg, Haidar et al., 2016; Piechota et al., 2012), and fixed dosages were within the range of 2–10 mg/kg (Georgiou et al., 2016; Roohbakhsh et al., 2021).

In studies reporting no significant changes in locomotion following METH withdrawal, the dosing regimen lasted longer, spanning from 7 days to 25 days (Henry et al., 2013; Gonzalez et al., 2014; Yan et al., 2019; Hosseini et al., 2021; Ghavimi et al., 2022). One study employed an escalating dose (ranging from 0.1 to 4 mg/kg, Henry et al., 2013), while fixed dosages typically ranged within 1–2 mg/kg (Gonzalez et al., 2014; Yan et al., 2019; Hosseini et al., 2021; Ghavimi et al., 2022).

Both sets of studies primarily assessed the animals’ behavior during the light phase. Studies reporting no significant changes during METH withdrawal assessed locomotion from day 1 to day 60 (Mandillo et al., 2003; Henry et al., 2013; Gonzalez et al., 2014; Yan et al., 2019; Hosseini et al., 2021; Ghavimi et al., 2022), while studies reporting hyperlocomotion assessed locomotion specifically from day 1 to day 12 during the abstinence period (Piechota et al., 2012; Georgiou et al., 2016; Haidar et al., 2016; Roohbakhsh et al., 2021).

Two studies investigated locomotion during abstinence from AMPH in mice. Haj-Mirzaian et al. (2018) found hyperlocomotion in NMRI mice after 5 days of 5 mg/kg AMPH administrations. However, Mandillo et al. (2003) reported no significant changes in locomotion following AMPH withdrawal. This discrepancy might be due to the lower AMPH dose used by Mandillo et al. (2.5 mg/kg) compared to Haj-Mirzaian et al. Additionally, Mandillo et al. used a different mouse strain (CD1) than Haj-Mirzaian et al. (NMRI) (Table 9).

**TABLE 9:** Hyperlocomotion *versus* no changes in locomotion: mice.

References	Strain/Species	Gender	Drug	Duration of drug Administration	Frequency of administration	Dosage	Light/dark phase	Withdrawal
Hyperlocomotion								
Georgiou et al (2016)	Mice C57BL/6J	Male	METH	10 days	1 time	2 mg/kg	Light	D7
Haidar et al (2016)	Mice Rln3KO	Female	METH	10 days	1 time	ESC 2–6 mg/kg	Light	D9
Roohbakhsh et al. (2021)	Mice	Male	METH	1 day	4 times 2 h interval	10 mg/kg	-	D1
Piechota et al. (2012)	Mice C57BL/6J	Male	METH	12 days	2 times	ESC 2–8 mg/kg	-	D12
Haj-Mirzaian et al. (2018)	Mice NMRI	Male	AMPH	5 days	1 time	5 mg/kg	-	D1
No significant changes in locomotion								
Henry et al. (2013)	Mice Gp120tg	Male and female	METH	25 days	3 times	0.1–4 mg/kg (11 days) +6 mg/kg (4x/day)	Light	D7
Yan et al (2019)	Mice C57BL/6J	Male	METH	7 days	1 time	1 mg/kg	Light	D46, 53, 60
Gonzalez et al. (2014)	Mice C57BL/6J	Male	METH	7 days	1 time	1 mg/kg	Light	D1-3
Ghavimi et al. (2022)	Mice BACB/c	Male	METH	14 days	2 times	2 mg/kg	Light	D10
Hosseini et al. (2021)	Mice NMRI	Male	METH	14 days	2 times	2 mg/kg	Light	D12
Mandillo et al. (2003)	Mice CD1	Male	AMPH	5 days	1 time	2.5 mg/kg	Light	D1

## 4 Discussion

In our systematic review, we observed that mice, primarily those withdrawn from repeated METH administrations, displayed increased horizontal activity compared to control animals. On the other hand, rats withdrawn from repeated AMPH administrations exhibited reduced horizontal activity. This contradiction might be at least partly due to the time of day when locomotor activity was measured. As rodents are nocturnal creatures, being more active during the dark phase, the observed decrease in activity among rats could be due to their higher baseline activity during that phase. In line with this, in Forced Swim Test (FST), rats displayed less escape-oriented behavior, had lower levels of stress markers and lesser serotonin turnover in amygdala and frontal cortex when tested at night compared to day time. The results suggest that rats might be better able to cope with the stress of the test during dark phase compared to the day (Kelliher et al., 2000). Alternatively, a notable decrease in dopamine levels has been documented during the dark phase of AMPH withdrawal. This decrease might lead to reduced spontaneous movement, particularly during the night-time, without impacting spontaneous activity or dopamine turnover during daylight hours (Crippens et al., 1993). In addition, the increase in activity among mice might also be influenced by their lower baseline activity during the light phase. Furthermore, assessment duration also could have influenced the activity level. We found that studies reporting hyperlocomotion had an average assessment time of 38.3 min, while those reporting hypolocomotion had an average assessment time of 460.83 min. During longer assessment durations, animals might become accustomed to the study environment, resulting in decreased exploration or movement. However, it is also important to take note of the individual differences in animals responding to environmental stimuli. For instance, Klejbor et al. (2013) found that switching from light to dark increased activity in highly reactive (HR) rats but had no effect on low reactive (LR) rats. The finding highlights inherent differences in activity level among rodents during light/dark phase, depending on their natural tendency to explore or habituate. Moreover, studies using mice have reported an increase in locomotion in the open field (Valentinuzzi et al., 2000). Whereas, when observed in EPM, there was no significant effect of light/dark cycle manipulation on the locomotor activity of mice (Clénet, et al., 2006). The findings suggest that light influences activity level in some behavioural apparatus, such as open field, but not necessarily others. Therefore, multiple factors including the time-of-day assessment conducted, duration of assessment and type of behavioral apparatus used can contribute to the observed discrepancies in the activity level during the abstinence period.

The distinct effects of METH and AMPH withdrawal on animal locomotion is intriguing, with METH-withdrawn animals commonly displaying hyperlocomotion and AMPH-withdrawn animals exhibiting reduced locomotor activity. METH and AMPH interact with the dopamine transporter (DAT), a key target for psychostimulants. DAT plays a crucial role in clearing synaptic dopamine, affecting the strength and duration of dopaminergic signalling. Both AMPH and METH act as substrates for DAT, competitively hindering dopamine uptake (Hall et al., 2008). METH, reported to be a more potent and longer-lasting stimulant than AMPH at similar doses (National Institute on Drug Abuse, 2006), exhibits three times greater inhibition of dopamine uptake in synaptosomes compared to AMPH (Rothman et al., 2001). Moreover, in cells expressing DAT, METH more effectively triggers dopamine release than AMPH (Eshleman et al., 1994). However, contradictory findings exist (John and Jones, 2007), alongside reports indicating no discernible differences in the effects of these two stimulants (Johnson et al., 1998). In the presence of salient stimuli, METH demonstrates greater potency in increasing overall locomotor activity compared to AMPH. However, in the absence of such stimuli, their potency appears comparable (Hall et al., 2008). During abstinence, METH’s absence might lead to a more pronounced decrease in extracellular dopamine levels. Consequently, hyperlocomotion may emerge as an attempt to compensate for and restore depleted dopamine signalling, a response more noticeable with METH. However, previous studies have suggested that AMPH induces greater locomotor activity compared to METH by further stimulating activity through glutamate (GLU) release in the nucleus accumbens upon acute dosing (Shoblock et al., 2003). Repeated METH administration also has been associated with a hyperglutamatergic state involving the metabotropic glutamate receptor subtype 5 (mGlu5) in the striatum (Szumlinski et al., 2017). In addition, pharmacological antagonism of mGlu5 reduces METH-induced locomotor hyperactivity (Wright et al., 2016). Currently, the differences in the effects of repeated METH and AMPH administrations on glutamatergic activity are not clear.

While studies comparing the effects of AMPH and METH in the past have primarily been conducted within the same species of rodents, this systematic review revealed differential effects between rats and mice. The vesicular monoamine transporter 2 (VMAT2), a presynaptic protein crucial for the packaging and subsequent release of dopamine and other monoamines. Notably, rat striatal vesicles exhibit a higher abundance of VMAT2 protein compared to mouse vesicles (Staal et al., 2000). This highlights potential species-specific differences in dopamine regulation. Studies employing targeted manipulation of VMAT2 gene expression levels have revealed altered animals’ sensitivity to psychostimulants (Wang et al., 1997; König et al., 2020). Adult mice with reduced VMAT2 gene level expressed 25% lesser striatal dopamine content, and 40% of reduced extracellular dopamine level compared to wild type mice (Wang et al., 1997). Upon acute administration of cocaine or AMPH, mice with reduced VMAT2 expression displayed significantly higher locomotion compared to wild type mice (Wang et al., 1997; König et al., 2020). Whereas, during repeated cocaine administration, these mice showed no increase in locomotion on D8 of the treatment compared to the drug-induced enhanced locomotion seen on D1 (Wang et al., 1997), suggests a complex shift in sensitivity with continued drug exposure. In addition, studies have demonstrated that VMAT2 confers neuroprotection against METH toxicity in mice overexpressing VMAT2 (Lohr et al., 2015). This is particularly relevant in light of the observation that repeated administrations of high doses of METH lead to a decrease in VMAT2 and dopamine protein levels within the striatum (Eyerman and Yamamoto, 2007). Therefore, it is likely that the observed discrepancies in the locomotor activity may also arise from a combined effect of differential expression of VMAT2 towards the sensitivity and toxicity of METH or AMPH.

The route of administration, in addition to potency, has been shown to modulate behavioral responses to METH and AMPH. Based on the data presented in this review, pre-treatment with intraperitoneal METH generally increased locomotor activity during the abstinence period compared to the subcutaneous route. The administration of varying doses of METH, promoting locomotion (0.3 and 1 mg/kg) and stereotypy (3 mg/kg) via intraperitoneal and subcutaneous routes in Sprague Dawley (SD) rats resulted in distinctive outcomes. The highest total locomotor activity occurred notably after intraperitoneal administration at the highest dose (3 mg/kg). Conversely, the most pronounced stereotypy was observed following the highest subcutaneous dose. Furthermore, subcutaneous METH exhibited prolonged locomotor effects compared to the intraperitoneal route, despite no difference in the elimination half-life of METH between the routes. Subcutaneous administration led to a higher area under the curve for METH exposure, indicative of a slower absorption rate and sustained release. This route also displayed elevated peak concentrations of both METH and its metabolite (AMPH) compared to intraperitoneal administration (Gentry et al., 2004). Previous reports have consistently associated heightened stereotypy with higher subcutaneous METH doses, for instance profound oral stereotypy following 4.42 mg/kg subcutaneous doses of METH (Segal and Kuchenski, 1997). Stereotypy involves behaviors such as ambulation, inactive rearing, head bobbing, continuous biting or licking, circling, and continuous sniffing. As stereotypy intensifies, locomotor activity diminishes. The inverse relationship between locomotor activity and stereotypy suggests a potential explanation for reduced or no significant changes at high METH doses. The absorption of subcutaneous METH into the bloodstream occurs at a slower rate than the intraperitoneal route, resulting in a 100% bioavailability and prolonged drug effects. Conversely, intraperitoneal METH absorbs more rapidly but encounters hepatic first-pass metabolism, restricting the absorbed dose (with a bioavailability of 58%) (Gentry et al., 2004). Intraperitoneal METH administration modifies the concentration-time profile of METH and AMPH through hepatic first-pass metabolism (Sakai et al., 1983). This metabolic alteration, favoring increased AMPH formation, appears to reduce overall exposure to METH, thereby shifting the response from stereotypy to heightened locomotor effects (Gentry et al., 2004). This could possibly elucidate the heightened distance travelled by mice pre-treated intraperitoneally with METH compared to subcutaneous route.

Variations in animal strains, genotypes, gender, and age can influence the observed behavioral alterations during drug withdrawal. For example, studies indicate that C57BL/6J mice display heightened locomotor activity to AMPH (Zocchi et al., 1997; Ralph et al., 2001) and increased mesoaccumbens dopamine release compared to other mouse strains (Zocchi et al., 1997). Evaluation of striatal dopamine and metabolite levels in adult male Spontaneously Hypertensive Rats (SHR), Wistar Kyoto (WK), and SD rats showed no notable differences in baseline dopamine, homovanillic acid (HVA), and 5-HIAA (hydroxyindoleacetic acid) levels. However, WK rats exhibited lower baseline 3,4-Dihydroxyphenylacetic acid (DOPAC) levels compared to SD, hinting at potential alterations in dopamine turnover within this strain. Following an acute injection of AMPH (2 mg/kg, i. p), significant changes in DA, DOPAC, HVA, and 5-HIAA levels were observed across all strains, indicating a uniform response to the stimulant’s immediate effects (Ferguson et al., 2003). AMPH administration did not impact mean adjusted delay in any of these strains (Wooters and Bardo, 2011). Additionally, compared to WK, SD rats displayed reduced 50-kHz ultrasonic vocalization following acute AMPH administration (Manduca et al., 2014), suggesting the possibility of distinct AMPH withdrawal profiles across different strains of rats.

When exposed to METH, both adult and adolescent C57BL/6J mice exhibited dopamine losses in the striatum, while adolescent DBA/2 and 129S6SvEv mice showed different responses compared to their adult counterparts (Good et al., 2011). Compared to C57BL/6J mice, dd mice exhibited heightened susceptibility to repeated METH administration despite both strains displaying increased ambulatory activity in response to the drug. In dd mice, reductions in both 3H-spiperone binding sites (associated with D2 receptors) and 3H-WB4101 binding sites (associated with the dopamine transporter) were observed in the striatum, cortex, and hippocampus. Conversely, C57BL/6J mice exhibited reduced binding sites only for 3H-WB4101 in these regions, indicating strain-specific variations in METH-induced neurochemical changes (Hayashi et al., 1987). These variations may link the differential effects of METH in these strains to distinct regional sensitivities to the drug. BALB/c mice demonstrated higher levels of homovanillic acid (HVA) and HVA/dopamine turnover in the striatum and frontal cortex following acute METH dosing (8 mg/kg, s. c), indicating increased dopamine release and potential neurotoxicity, suggesting heightened sensitivity to METH’s adverse effects compared to C57BL/6J mice (Halladay et al., 2003). However, another study reported conflicting results, indicating that C57BL/6J mice experienced more pronounced dopamine depletion than BALB/c mice, with serotonin depletion occurring solely in male BALB/c mice compared to C57BL/6J mice. Additionally, male C57BL/6J mice exhibited greater dopamine depletion than females, while BALB/c mice did not show sex-based differences following METH treatment (Yu and Liao, 2000). These findings suggest sex-strain disparities in susceptibility to METH-induced effects. While dopamine is central to the reinforcing effects of AMPH, withdrawal symptoms are influenced by other brain regions and neurotransmitters such as serotonin and glutamate. Investigating how different strains experience changes in these and other neuromodulators could provide valuable understanding of their involvement in stimulant-induced increased sensitivity to movement during periods of abstinence.

Drug administration is stressful, especially with increased injections, as seen in studies using escalating schedules. This heightened injection stress in animals may lead to cross-sensitization, potentially resulting in more pronounced locomotor reactions (Russig et al., 2005). Our systematic review’s findings suggest that in studies reporting AMPH-withdrawal induced hypolocomotion, rats generally received a higher total number of injections compared to AMPH-withdrawn rats showing no significant changes in horizontal activity. Conversely, in mice, METH-withdrawn animals with increased locomotion (intraperitoneal route: 4–24) received fewer injections overall compared to METH-withdrawn animals displaying no significant changes in locomotion (intraperitoneal: 7–28, subcutaneous (1 study): 86). These observations indicate insufficient evidence to conclusively link injection stress with stimulant-induced changes in rodents’ horizontal activity during abstinence.

It is crucial to acknowledge that different animal strains or genotypes may respond variably to stress, influencing the behavioral outcomes (O’Mahony et al., 2010; Marchette et al., 2018). Based on the current body of literature, it seems that rats may be more vulnerable to withdrawal stress, potentially accounting for observed differences in locomotion during AMPH withdrawal. Several studies consistently show heightened anxiety-like behavior (Vuong et al., 2010; Tu et al., 2014) and increased activity in the HPA axis (Bray et al., 2016) during AMPH withdrawal in rats. Conversely, findings related to withdrawal stress in mice are less robust. There are even reports stating that withdrawal from repeated AMPH pre-treatment did not alter anxiety-like behavior in mice (Fukushiro et al., 2011). Consistent with this, several studies suggest that mice may exhibit greater resilience to stress-induced anxiety. For instance, exposure to single prolonged stress failed to induce anxiety-like behavior in mice (You et al., 2021), and adult mice do not seem to experience lasting effects following chronic stress (Barnum et al., 2012). Furthermore, mice have been noted to display reduced anxiety-like behavior in the elevated plus maze, potentially attributed to elevated neuropeptide Y levels in the amygdala, indicating a potential resistance to stress-induced anxiety (Nguyen et al., 2009). While not entirely impervious to stress, mice may showcase enhanced adaptability and flexibility in unfamiliar environments.

## 5 Conclusion

Based on the findings from studies involving mice and rats included in this review, genetic diversity and species difference can significantly impact METH and AMPH withdrawal responses. However, a majority (more than 50%) of the reviewed studies reported no significant difference in the animals’ locomotion during the abstinence period. Several factors might contribute to this, such as heterogeneity in study designs and differences in withdrawal time point of assessments. Despite the insignificant results, understanding the potential for species-specific responses remains crucial as this can help researchers design their studies accordingly. Based on the reviewed studies, the findings suggest METH withdrawal primarily leads to hyperlocomotion, while withdrawal from AMPH appears to induce hypolocomotion. Translating these preclinical findings to human population is vital in understanding how prolonged METH or AMPH use leads to physical dependence during the abstinence period. Exploring the potential differences in the mechanism of action of METH and AMPH could ultimately lead to development of more targeted therapy strategies in substance use disorder.

## Data Availability

The original contributions presented in the study are included in the article/Supplementary Material, further inquiries can be directed to the corresponding author.
